# Infant mortality rates regressed against number of vaccine doses routinely given: Is there a biochemical or synergistic toxicity?

**DOI:** 10.1177/0960327111407644

**Published:** 2011-09

**Authors:** Neil Z Miller, Gary S Goldman

**Keywords:** infant mortality rates, sudden infant death, SIDS, immunization schedules, childhood vaccines, drug toxicology, synergistic effects, linear regression model

## Abstract

The infant mortality rate (IMR) is one of the most important indicators of the socio-economic well-being and public health conditions of a country. The US childhood immunization schedule specifies 26 vaccine doses for infants aged less than 1 year—the most in the world—yet 33 nations have lower IMRs. Using linear regression, the immunization schedules of these 34 nations were examined and a correlation coefficient of *r* = 0.70 (*p* < 0.0001) was found between IMRs and the number of vaccine doses routinely given to infants. Nations were also grouped into five different vaccine dose ranges: 12–14, 15–17, 18–20, 21–23, and 24–26. The mean IMRs of all nations within each group were then calculated. Linear regression analysis of unweighted mean IMRs showed a high statistically significant correlation between increasing number of vaccine doses and increasing infant mortality rates, with *r* = 0.992 (*p* = 0.0009). Using the Tukey-Kramer test, statistically significant differences in mean IMRs were found between nations giving 12–14 vaccine doses and those giving 21–23, and 24–26 doses. A closer inspection of correlations between vaccine doses, biochemical or synergistic toxicity, and IMRs is essential.

## Introduction

The infant mortality rate (IMR) is one of the most important measures of child health and overall development in countries. Clean water, increased nutritional measures, better sanitation, and easy access to health care contribute the most to improving infant mortality rates in unclean, undernourished, and impoverished regions of the world.^1–3^ In developing nations, IMRs are high because these basic necessities for infant survival are lacking or unevenly distributed. Infectious and communicable diseases are more common in developing countries as well, though sound sanitary practices and proper nutrition would do much to prevent them.^1^

The World Health Organization (WHO) attributes 7 out of 10 childhood deaths in developing countries to five main causes: pneumonia, diarrhea, measles, malaria, and malnutrition—the latter greatly affecting all the others.^1^ Malnutrition has been associated with a decrease in immune function. An impaired immune function often leads to an increased susceptibility to infection.^2^ It is well established that infections, no matter how mild, have adverse effects on nutritional status. Conversely, almost any nutritional deficiency will diminish resistance to disease.^3^

Despite the United States spending more per capita on health care than any other country,^4^ 33 nations have better IMRs. Some countries have IMRs that are less than half the US rate: Singapore, Sweden, and Japan are below 2.80. According to the Centers for Disease Control and Prevention (CDC), “The relative position of the United States in comparison to countries with the lowest infant mortality rates appears to be worsening.”^5^

There are many factors that affect the IMR of any given country. For example, premature births in the United States have increased by more than 20% between 1990 and 2006. Preterm babies have a higher risk of complications that could lead to death within the first year of life.^6^ However, this does not fully explain why the United States has seen little improvement in its IMR since 2000.^7^

Nations differ in their immunization requirements for infants aged less than 1 year. In 2009, five of the 34 nations with the best IMRs required 12 vaccine doses, the least amount, while the United States required 26 vaccine doses, the most of any nation. To explore the correlation between vaccine doses that nations routinely give to their infants and their infant mortality rates, a linear regression analysis was performed.

## Methods and design

### Infant mortality

The infant mortality rate is expressed as the number of infant deaths per 1000 live births. According to the US Central Intelligence Agency (CIA), which keeps accurate, up-to-date infant mortality statistics throughout the world, in 2009 there were 33 nations with better infant mortality rates than the United States (Table 1).^8^ The US infant mortality rate of 6.22 infant deaths per 1000 live births ranked 34th.

**Table 1.** 2009 Infant mortality rates, top 34 nations^8^

Rank	Country	IMR
1	Singapore	2.31
2	Sweden	2.75
3	Japan	2.79
4	Iceland	3.23
5	France	3.33
6	Finland	3.47
7	Norway	3.58
8	Malta	3.75
9	Andorra	3.76
10	Czech Republic	3.79
11	Germany	3.99
12	Switzerland	4.18
13	Spain	4.21
14	Israel	4.22
15	Liechtenstein	4.25
16	Slovenia	4.25
17	South Korea	4.26
18	Denmark	4.34
19	Austria	4.42
20	Belgium	4.44
21	Luxembourg	4.56
22	Netherlands	4.73
23	Australia	4.75
24	Portugal	4.78
25	United Kingdom	4.85
26	New Zealand	4.92
27	Monaco	5.00
28	Canada	5.04
29	Ireland	5.05
30	Greece	5.16
31	Italy	5.51
32	San Marino	5.53
33	Cuba	5.82
34	United States	6.22

CIA. Country comparison: infant mortality rate (2009). *The World Factbook.* www.cia.gov (Data last updated 13 April 2010).^8^

### Immunization schedules and vaccine doses

A literature review was conducted to determine the immunization schedules for the United States and all 33 nations with better IMRs than the United States.^9,10^ The total number of vaccine doses specified for infants aged less than 1 year was then determined for each country (Table 2). A vaccine dose is an exact amount of medicine or drug to be administered. The number of doses a child receives should not be confused with the number of ‘vaccines' or ‘injections' given. For example, DTaP is given as a single injection but contains three separate vaccines (for diphtheria, tetanus, and pertussis) totaling three vaccine doses.

**Table 2.** Summary of International Immunization Schedules: vaccines recommended/required prior to one year of age in 34 nations

Nation	Vaccines prior to one year of age	Total^b^ doses	Group (range of doses)
Sweden	DTaP (2), Polio (2), Hib (2), Pneumo (2)	12	1 (12–14)
Japan	DTaP (3), Polio (2), BCG	12	
Iceland	DTaP (2), Polio (2), Hib (2), MenC (2)	12	
Norway	DTaP (2), Polio (2), Hib (2), Pneumo (2)	12	
Denmark	DTaP (2), Polio (2), Hib (2), Pneumo (2)	12	
Finland	DTaP (2), Polio (2), Hib (2), Rota (3)	13	
Malta	DTaP (3), Polio (3), Hib (3)	15	2 (15–17)
Slovenia	DTaP (3), Polio (3), Hib (3)	15	
South Korea	DTaP (3), Polio (3), HepB (3)	15	
Singapore	DTaP (3), Polio (3), HepB (3), BCG, Flu	17	
New Zealand	DTaP (3), Polio (3), Hib (2), HepB (3)	17	
Germany	DTaP (3), Polio (3), Hib (3), Pneumo (3)	18	3 (18–20)
Switzerland	DTaP (3), Polio (3), Hib (3), Pneumo (3)	18	
Israel	DTaP (3), Polio (3), Hib (3), HepB (3)	18	
Liechtenstein^a^	DTaP (3), Polio (3), Hib (3), Pneumo (3)	18	
Italy	DTaP (3), Polio (3), Hib (3), HepB (3)	18	
San Marino^a^	DTaP (3), Polio (3), Hib (3), HepB (3)	18	
France	DTaP (3), Polio (3), Hib (3), Pneumo (2), HepB (2)	19	
Czech Republic	DTaP (3), Polio (3), Hib (3), HepB (3), BCG	19	
Belgium	DTaP (3), Polio (3), Hib (3), HepB (3), Pneumo (2)	19	
United Kingdom	DTaP (3), Polio (3), Hib (3), Pneumo (2), MenC (2)	19	
Spain	DTaP (3), Polio (3), Hib (3), HepB (3), MenC (2)	20	
Portugal	DTaP (3), Polio (3), Hib (3), HepB (3), MenC (2), BCG	21	4 (21–23)
Luxembourg	DTaP (3), Polio (3), Hib (3), HepB (2), Pneumo (3), Rota (3)	22	
Cuba	DTaP (3), Polio (3), Hib (3), HepB (4), MenBC (2), BCG	22	
Andorra^a^	DTaP (3), Polio (3), Hib (3), HepB (3), Pneumo (3), MenC (2)	23	
Austria	DTaP (3), Polio (3), Hib (3), HepB (3), Pneumo (3), Rota (2)	23	
Ireland	DTaP (3), Polio (3), Hib (3), HepB (3), Pneumo (2), MenC (2), BCG	23	
Greece	DTaP (3), Polio (3), Hib (3), HepB (3), Pneumo (3), MenC (2)	23	
Monaco^a^	DTaP (3), Polio (3), Hib (3), HepB (3), Pneumo (3), HepA, BCG	23	
Netherlands	DTaP (4), Polio (4), Hib (4), Pneumo (4)	24	5 (24–26)
Canada	DTaP (3), Polio (3), Hib (3), HepB (3), Pneumo (3), MenC (2), Flu	24	
Australia	DTaP (3), Polio (3), Hib (3), HepB (4), Pneumo (3), Rota (2)	24	
United States	DTaP (3), Polio (3), Hib (3), HepB (3), Pneumo (3), Rota (3), Flu (2)	26	

^a^ These four nations were excluded from the analysis because they had fewer than five infant deaths.

^b^ DTaP is administered as a single shot but contains three separate vaccines (for diphtheria, tetanus, and pertussis). Thus, DTaP given three times in infancy is equivalent to nine vaccine doses. Immunization schedules are for 2008–2009.^9,10^

### Nations organized into data pairs

The 34 nations were organized into data pairs consisting of total number of vaccine doses specified for their infants and IMRs. Consistent with biostatistical conventions, four nations—Andorra, Liechenstein, Monaco, and San Marino—were excluded from the dataset because they each had fewer than five infant deaths, producing extremely wide confidence intervals and IMR instability. The remaining 30 (88%) of the data pairs were then available for analysis.

### Nations organized into groups

Nations were placed into the following five groups based on the number of vaccine doses they routinely give their infants: 12–14, 15–17, 18–20, 21–23, and 24–26 vaccine doses. The unweighted IMR means of all nations as a function of the number of vaccine doses were analyzed using linear regression. The Pearson correlation coefficient (*r*) and coefficient of determination (*r* ^2^) were calculated using GraphPad Prism, version 5.03 (GraphPad Software, San Diego, CA, USA, www.graphpad.com). Additionally, the *F* statistic and corresponding *p* values were computed to test if the best fit slope was statistically significantly non-zero. The Tukey-Kramer test was used to determine whether or not the mean IMR differences between the groups were statistically significant. Following the one-way ANOVA (analysis of variance) results from the Tukey-Kramer test, a post test for the overall linear trend was performed.

## Results

### Nations organized into data pairs

**Figure 1.**
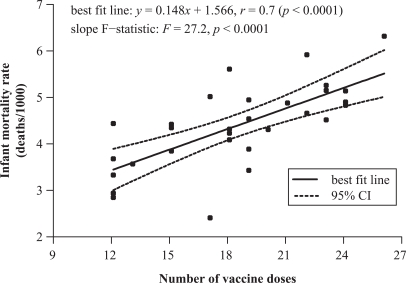
2009 Infant mortality rates and number of vaccine doses for 30 nations.

A scatter plot of each of the 30 nation’s IMR versus vaccine doses yielded a linear relationship with a correlation coefficient of 0.70 (95% CI, 0.46–0.85) and *p* < 0.0001 providing evidence of a positive correlation: IMR and vaccine doses tend to increase together. The *F* statistic applied to the slope [0.148 (95% CI, 0.090–0.206)] is significantly non-zero, with *F* = 27.2 (*p* < 0.0001; Figure 1).

### Nations organized into groups

**Figure 2.**
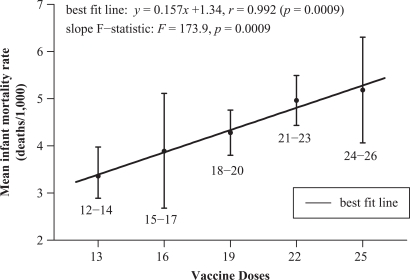
2009 Mean infant mortality rates and mean number of vaccine doses (five categories).

The unweighted mean IMR of each category was computed by simply summing the IMRs of each nation comprising a group and dividing by the number of nations in that group. The IMRs were as follows: 3.36 (95% CI, 2.74–3.98) for nations specifying 12–14 doses (mean 13 doses); 3.89 (95% CI, 2.68–5.12) for 15–17 doses (mean 16 doses); 4.28 (95% CI, 3.80–4.76) for 18–20 doses (mean 19 doses); 4.97 (95% CI, 4.44–5.49) for 21–23 doses (mean 22 doses); 5.19 (95% CI, 4.06–6.31) for 24-26 doses (mean 25 doses; Figure 2). Linear regression analysis yielded an equation of the best fit line, *y* = 0.157*x* + 1.34 with *r* = 0.992 (*p* = 0.0009) and *r* ^2^ = 0.983. Thus, 98.3% of the variation in mean IMRs is explained by the linear model. Again, the *F* statistic yielded a significantly non-zero slope, with *F* = 173.9 (*p* = 0.0009).

The one-way ANOVA using the Tukey-Kramer test yielded *F* = 650 with *p* = 0.001, indicating the five mean IMRs corresponding to the five defined dose categories are significantly different (*r* ^2^ = 0.510). Tukey’s multiple comparison test found statistical significance in the differences between the mean IMRs of those nations giving 12–14 vaccine doses and (a) those giving 21–23 doses (1.61, 95% CI, 0.457–2.75) and (b) those giving 24–26 doses (1.83, 95% CI, 0.542–3.11).

## Discussion

### Basic necessities for infant survival

It is instructive to note that many developing nations require their infants to receive multiple vaccine doses and have national vaccine coverage rates (a percentage of the target population that has been vaccinated) of 90% or better, yet their IMRs are poor. For example, Gambia requires its infants to receive 22 vaccine doses during infancy and has a 91%–97% national vaccine coverage rate, yet its IMR is 68.8. Mongolia requires 22 vaccine doses during infancy, has a 95%–98% coverage rate, and an IMR of 39.9.^8,9^ These examples appear to confirm that IMRs will remain high in nations that cannot provide clean water, proper nutrition, improved sanitation, and better access to health care. *As developing nations improve in all of these areas a critical threshold will eventually be reached where further reductions of the infant mortality rate will be difficult to achieve because most of the susceptible infants that could have been saved from these causes would have been saved.* Further reductions of the IMR must then be achieved in areas outside of these domains. As developing nations ascend to higher socio-economic living standards, a closer inspection of all factors contributing to infant deaths must be made.

### Crossing the socio-economic threshold

It appears that at a certain stage in nations' movement up the socio-economic scale—after the basic necessities for infant survival (proper nutrition, sanitation, clean water, and access to health care) have been met—a counter-intuitive relationship occurs between the number of vaccines given to infants and infant mortality rates: nations with higher (worse) infant mortality rates give their infants, on average, more vaccine doses. This positive correlation, derived from the data and demonstrated in Figures 1 and 2, elicits an important inquiry: are some infant deaths associated with over-vaccination?

### A closer inspection of infant deaths

Many nations adhere to an agreed upon International Classification of Diseases (ICD) for grouping infant deaths into 130 categories.^11–13^ Among the 34 nations analyzed, those that require the most vaccines tend to have the worst IMRs. Thus, we must ask important questions: is it possible that some nations are requiring too many vaccines for their infants and the additional vaccines are a toxic burden on their health? Are some deaths that are listed within the 130 infant mortality death categories really deaths that are associated with over-vaccination? Are some vaccine-related deaths hidden within the death tables?

### Sudden infant death syndrome (SIDS)

Prior to contemporary vaccination programs, ‘Crib death’ was so infrequent that it was not mentioned in infant mortality statistics. In the United States, national immunization campaigns were initiated in the 1960s when several new vaccines were introduced and actively recommended. For the first time in history, most US infants were required to receive several doses of DPT, polio, measles, mumps, and rubella vaccines.^14^ Shortly thereafter, in 1969, medical certifiers presented a new medical term—sudden infant death syndrome.^15,16^ In 1973, the National Center for Health Statistics added a new cause-of-death category—for SIDS—to the ICD. SIDS is defined as the sudden and unexpected death of an infant which remains unexplained after a thorough investigation. Although there are no specific symptoms associated with SIDS, an autopsy often reveals congestion and edema of the lungs and inflammatory changes in the respiratory system.^17^ By 1980, SIDS had become the leading cause of postneonatal mortality (deaths of infants from 28 days to one year old) in the United States.^18^

In 1992, to address the unacceptable SIDS rate, the American Academy of Pediatrics initiated a ‘Back to Sleep’ campaign, convincing parents to place their infants supine, rather than prone, during sleep. From 1992 to 2001, the postneonatal SIDS rate dropped by an average annual rate of 8.6%. However, other causes of sudden unexpected infant death (SUID) increased. For example, the postneonatal mortality rate from ‘suffocation in bed’ (ICD-9 code E913.0) increased during this same period at an average annual rate of 11.2%. The postneonatal mortality rate from ‘suffocation-other’ (ICD-9 code E913.1-E913.9), ‘unknown and unspecified causes' (ICD-9 code 799.9), and due to ‘intent unknown’ in the External Causes of Injury section (ICD-9 code E980-E989), all increased during this period as well.^18^ (In Australia, Mitchell et al. observed that when the SIDS rate decreased, deaths attributed to asphyxia increased.^19^ Overpeck et al. and others, reported similar observations.)^20,21^

**Figure 3.**
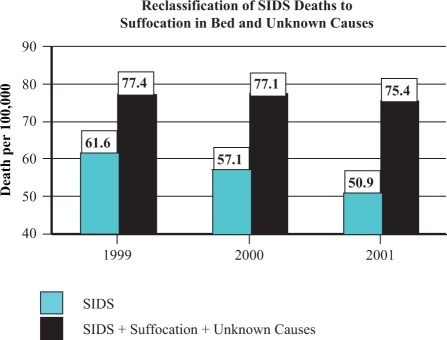
Reclassification of sudden infant death syndrome (SIDS) deaths to suffocation in bed and unknown causes. The postneonatal SIDS rate appears to have declined from 61.6 deaths (per 100,000 live births) in 1999 to 50.9 in 2001. However, during this period there was a significant increase in postneonatal deaths attributed to suffocation in bed and due to unknown causes. When these sudden unexpected infant deaths (SUIDs) are combined with SIDS deaths, the total SIDS rate remains relatively stable, resulting in a non-significant decline.

A closer inspection of the more recent period from 1999 to 2001 reveals that the US postneonatal SIDS rate continued to decline, but *there was no significant change in the total postneonatal mortality rate.* During this period, the number of deaths attributed to ‘suffocation in bed’ and ‘unknown causes,’ increased significantly. According to Malloy and MacDorman, “If death-certifier preference has shifted such that previously classified SIDS deaths are now classified as ‘suffocation,’ the inclusion of these suffocation deaths and unknown or unspecified deaths with SIDS deaths then accounts for about 90 percent of the decline in the SIDS rate observed between 1999 and 2001 and results in a non-significant decline in SIDS”^18^ (Figure 3).

### Is there evidence linking SIDS to vaccines?

Although some studies were unable to find correlations between SIDS and vaccines,^22–24^ there is some evidence that a subset of infants may be more susceptible to SIDS shortly after being vaccinated. For example, Torch found that two-thirds of babies who had died from SIDS had been vaccinated against DPT (diphtheria–pertussis–tetanus toxoid) prior to death. Of these, 6.5% died within 12 hours of vaccination; 13% within 24 hours; 26% within 3 days; and 37%, 61%, and 70% within 1, 2, and 3 weeks, respectively. Torch also found that unvaccinated babies who died of SIDS did so most often in the fall or winter while vaccinated babies died most often at 2 and 4 months—the same ages when initial doses of DPT were given to infants. He concluded that DPT “may be a generally unrecognized major cause of sudden infant and early childhood death, and that the risks of immunization may outweigh its potential benefits. A need for re-evaluation and possible modification of current vaccination procedures is indicated by this study.”^25^ Walker et al. found “the SIDS mortality rate in the period zero to three days following DPT to be 7.3 times that in the period beginning 30 days after immunization.”^26^ Fine and Chen reported that babies died at a rate nearly eight times greater than normal within 3 days after getting a DPT vaccination.^27^

Ottaviani et al. documented the case of a 3-month-old infant who died suddenly and unexpectedly shortly after being given six vaccines in a single shot: “Examination of the brainstem on serial sections revealed bilateral hypoplasia of the arcuate nucleus. The cardiac conduction system presented persistent fetal dispersion and resorptive degeneration. This case offers a unique insight into the possible role of hexavalent vaccine in triggering a lethal outcome in a vulnerable baby.” Without a full necropsy study in the case of sudden, unexpected infant death, at least some cases linked to vaccination are likely to go undetected.^28^

### Reclassified infant deaths

It appears as though some infant deaths attributed to SIDS may be vaccine related, perhaps associated with biochemical or synergistic toxicity due to over-vaccination. Some infants' deaths categorized as ‘suffocation’ or due to ‘unknown and unspecified causes' may also be cases of SIDS reclassified within the ICD. Some of these infant deaths may be vaccine related as well. This trend toward reclassifying ICD data is a great concern of the CDC “because inaccurate or inconsistent cause-of-death determination and reporting hamper the ability to monitor national trends, ascertain risk factors, and design and evaluate programs to prevent these deaths.”^29^ If some infant deaths are vaccine related and concealed within the various ICD categories for SUIDs, is it possible that other vaccine-related infant deaths have also been reclassified?

Of the 34 nations that have crossed the socio-economic threshold and are able to provide the basic necessities for infant survival—clean water, nutrition, sanitation, and health care—several require their infants to receive a relatively high number of vaccine doses and have relatively high infant mortality rates. These nations should take a closer look at their infant death tables to determine if some fatalities are possibly related to vaccines though reclassified as other causes. Of course, all SUID categories should be re-inspected. Other ICD categories may be related to vaccines as well. For example, a new live-virus orally administered vaccine against rotavirus-induced diarrhea—Rotarix^®^—was licensed by the European Medicine Agency in 2006 and approved by the US Food and Drug Administration (FDA) in 2008. However, in a clinical study that evaluated the safety of the Rotarix vaccine, *vaccinated babies died at a higher rate than non-vaccinated babies*—mainly due to a statistically significant increase in pneumonia-related fatalities.^30^ (One biologically plausible explanation is that natural rotavirus infection might have a protective effect against respiratory infection.)^31^ Although these fatalities appear to be vaccine related and raise a nation’s infant mortality rate, medical certifiers are likely to misclassify these deaths as pneumonia.

Several additional ICD categories are possible candidates for incorrect infant death classifications: unspecified viral diseases, diseases of the blood, septicemia, diseases of the nervous system, anoxic brain damage, other diseases of the nervous system, diseases of the respiratory system, influenza, and unspecified diseases of the respiratory system. All of these selected causes may be repositories of vaccine-related infant deaths reclassified as common fatalities. All nations—rich and poor, industrialized and developing—have an obligation to determine whether their immunization schedules are achieving their desired goals. Progress on reducing infant mortality rates should include monitoring vaccine schedules and medical certification practices to ascertain whether vaccine-related infant deaths are being reclassified as ordinary mortality in the ICD.

### How many infants can be saved with an improved IMR?

Slight improvements in IMRs can make a substantial difference. In 2009, there were approximately 4.5 million live births and 28,000 infant deaths in the United States, resulting in an infant mortality rate of 6.22/1000. If health authorities can find a way to reduce the rate by 1/1000 (16%), the United States would rise in international rank from 34th to 31st and about 4500 infants would be saved.

## Limitations of study and potential confounding factors

This analysis did not adjust for vaccine composition, national vaccine coverage rates, variations in the infant mortality rates among minority races, preterm births, differences in how some nations report live births, or the potential for ecological bias. A few comments about each of these factors are included below.

### Vaccine composition

This analysis calculated the total number of vaccine doses received by children but did not differentiate between the substances, or quantities of those substances, in each dose. Common vaccine substances include antigens (attenuated viruses, bacteria, toxoids), preservatives (thimerosal, benzethonium chloride, 2-phenoxyethanol, phenol), adjuvants (aluminum salts), additives (ammonium sulfate, glycerin, sodium borate, polysorbate 80, hydrochloric acid, sodium hydroxide, potassium chloride), stabilizers (fetal bovine serum, monosodium glutamate, human serum albumin, porcine gelatin), antibiotics (neomycin, streptomycin, polymyxin B), and inactivating chemicals (formalin, glutaraldehyde, polyoxyethylene). For the purposes of this study, all vaccine doses were equally weighted.

### Vaccine coverage rates

No adjustment was made for national vaccine coverage rates—a percentage of the target population that received the recommended vaccines. However, most of the nations in this study had coverage rates in the 90%–99% range for the most commonly recommended vaccines—DTaP, polio, hepatitis B, and Hib (when these vaccines were included in the schedule). Therefore, this factor is unlikely to have impacted the analyses.^9^

### Minority races

It has been argued that the US IMR is poor in comparison to many other nations because African–American infants are at greater risk of dying relative to White infants, perhaps due to genetic factors or disparities in living standards. However, in 2006 the US IMR for infants of all races was 6.69 and the IMR for White infants was 5.56.^13^ In 2009, this improved rate would have moved the United States up by just one rank internationally, from 34th place to 33rd place.^8^ In addition, the IMRs for Hispanics of Mexican descent and Asian–Americans in the United States are significantly lower than the IMR for Whites.^6^ Thus, diverse IMRs among different races in the Unites States exert only a modest influence over the United States' international infant mortality rank.

### Preterm births

Preterm birth rates in the United States have steadily increased since the early 1980s. (This rise has been tied to a greater reliance on caesarian deliveries, induced labor, and more births to older mothers.) Preterm babies are more likely than full-term babies to die within the first year of life. About 12.4% of US births are preterm. In Europe, the prevalence rate of premature birth ranges from 5.5% in Ireland to 11.4% in Austria. Preventing preterm births is essential to lower infant mortality rates. However, it is important to note that some nations such as Ireland and Greece, which have very low preterm birth rates (5.5% and 6%, respectively) compared to the United States, require their infants to receive a relatively high number of vaccine doses (23) and have correspondingly high IMRs. Therefore, reducing preterm birth rates is only part of the solution to reduce IMRs.^6,32^

### Differences in reporting live births

Infant mortality rates in most countries are reported using WHO standards, which do not include any reference to the duration of pregnancy or weight of the infant, but do define a ‘live birth’ as a baby born with any signs of life for any length of time.^12^ However, four nations in the dataset—France, the Czech Republic, the Netherlands, and Ireland—do not report live births entirely consistent with WHO standards. These countries add an additional requirement that live babies must also be at least 22 weeks of gestation or weigh at least 500 grams. If babies do not meet this requirement and die shortly after birth, they are reported as stillbirths. This inconsistency in reporting live births artificially lowers the IMRs of these nations.^32,33^ According to the CDC, “There are some differences among countries in the reporting of very small infants who may die soon after birth. However, it appears unlikely that differences in reporting are the primary explanation for the United States' relatively low international ranking.”^32^ Nevertheless, when the IMRs of France, the Czech Republic, the Netherlands, and Ireland were adjusted for known underreporting of live births and the 30 data pairs retested for significance, the correlation coefficient improved from 0.70 to 0.74 (95% CI, 0.52–0.87).

### Ecological bias

Ecological bias occurs when relationships among individuals are inferred from similar relationships observed among groups (or nations). Although most of the nations in this study had 90%–99% of their infants fully vaccinated, without additional data we do not know whether it is the vaccinated or unvaccinated infants who are dying in infancy at higher rates. However, respiratory disturbances have been documented in close proximity to infant vaccinations, and lethal changes in the brainstem of a recently vaccinated baby have been observed. Since some infants may be more susceptible to SIDS shortly after being vaccinated, and babies vaccinated against diarrhea died from pneumonia at a statistically higher rate than non-vaccinated babies, there is plausible biologic and causal evidence that the observed correlation between IMRs and the number of vaccine doses routinely given to infants should not be dismissed as ecological bias.

## Conclusion

The US childhood immunization schedule requires 26 vaccine doses for infants aged less than 1 year, the most in the world, yet 33 nations have better IMRs. Using linear regression, the immunization schedules of these 34 nations were examined and a correlation coefficient of 0.70 (*p* < 0.0001) was found between IMRs and the number of vaccine doses routinely given to infants. When nations were grouped into five different vaccine dose ranges (12–14, 15–17, 18–20, 21–23, and 24–26), 98.3% of the total variance in IMR was explained by the unweighted linear regression model. These findings demonstrate a counter-intuitive relationship: *nations that require more vaccine doses tend to have higher infant mortality rates*.

Efforts to reduce the relatively high US IMR have been elusive. Finding ways to lower preterm birth rates should be a high priority. However, preventing premature births is just a partial solution to reduce infant deaths. A closer inspection of correlations between vaccine doses, biochemical or synergistic toxicity, and IMRs, is essential. All nations—rich and poor, advanced and developing—have an obligation to determine whether their immunization schedules are achieving their desired goals.
